# Supplementary material for: Elizabethkingia anophelis: Physiologic and Transcriptomic Responses to Iron Stress
Source: Front Microbiol. 2020 May 7;11:804. doi: 10.3389/fmicb.2020.00804 (PMC7221216; doi:10.3389/fmicb.2020.00804)
Supplement: Supplementary file 4 [file Data_Sheet_1.zip › Figure S1.docx]

**Figure S1. The transcriptional start site (TSS) for gene of Elilysin2 (EAAG1_11027) determination and promoter prediction.** A) The TSS was determined as C, 29 bp upstream of the translation start site. Red font and underlined. The promoter motifs were highlighted with underline. B) The folding of the promoter regions was predicted with RNAfold (<http://rna.tbi.univie.ac.at)>.

*E. anophelis* grown in LB broth with high or low iron concentration respectively, to an OD600 of about 0.5. Cells were harvested by centrifugation at 4,000 g for 10 minutes at room temperature. Pellets were resuspended in 2 ml of RNAlater (Qiagen, USA) and stored at -80°C. Bacterial cell pellets stored at -80°C in RNAlater (25 ml) were thawed on ice, re-suspended and re-pelleted in 1 ml aliquots for 10 minutes at 4,000 g in a microcentrifuge. The supernatant was removed and 200 μl bacterial lysis buffer (30 mM Tris HCl, pH 8.0, 1 mM EDTA plus 15 mg/ml lysozyme (Sigma, St Louis, MO, USA) and 15 μl proteinase K (20mg/ml; QIAGEN, Valencia, CA, USA) were added to each tube. Samples were incubated at room temperature for 10 minutes, and vortexed for 10 s before and every 2 minutes during the incubation. QIAGEN RLT Plus buffer (750 μl) supplemented with 1 % v/v beta-mercaptoethanol (Sigma) was added to each tube and vortexed briefly to mix. Analysis by 5’ rapid amplification of cDNA ends (5’RACE) was done by using the SMARTer RACE 5’/3’ kit (Clontech, CA) under the manipulation procedures recommended by the supplier. For Pheam, the first cDNA strand was done with a specific primer Walker188 which is based on hemolysin gene sequence (Table 2). The 5’-end region of interest was then amplified by using two sets of primers for regions of heamlysin in a nested PCR. In the first PCR, RT products were used as a template and amplified with primers Walker188 and UPM (Universal Primer Mix). The second PCR (nested PCR) was conducted with primers Walker189 and Nested UPM with the 10-fold dilution of the round 1 PCR product as the template (Table 2). The 5-RACE product with a size of ~450-bp were isolated, purified, ligated into the pGEM-T Easy vector, and sequenced. 5’RACE-PCR showed that the transcriptional start site is C. Immediate 29-bp upstream of the translational start site, there is a conserved promoter motif TTG-N19-TAnnTTTG (1). The promoter resembles the consensus promoter structure in other *Bacteroidetes*.

1. **Chen S, Bagdasarian M, Kaufman M, Walker E.** 2007. Characterization of strong promoters from an environmental *Flavobacterium hibernum* strain by using a green fluorescent protein-based reporter system. Appl Environ Microbiol **73:**1089 - 1100.
