# Supplementary material for: Elizabethkingia anophelis: Physiologic and Transcriptomic Responses to Iron Stress
Source: Front Microbiol. 2020 May 7;11:804. doi: 10.3389/fmicb.2020.00804 (PMC7221216; doi:10.3389/fmicb.2020.00804)
Supplement: Supplementary file 4 [file Data_Sheet_1.zip › Figure S2.docx]

**Figure S2. PCA analysis of the high and low iron RNAseq libraries.** The iron minus indicated low-iron cultures and iron plus indicated the high iron cultures.
