# Supplementary material for: Elizabethkingia anophelis: Physiologic and Transcriptomic Responses to Iron Stress
Source: Front Microbiol. 2020 May 7;11:804. doi: 10.3389/fmicb.2020.00804 (PMC7221216; doi:10.3389/fmicb.2020.00804)
Supplement: Supplementary file 4 [file Data_Sheet_1.zip › Figure S3.docx]

**
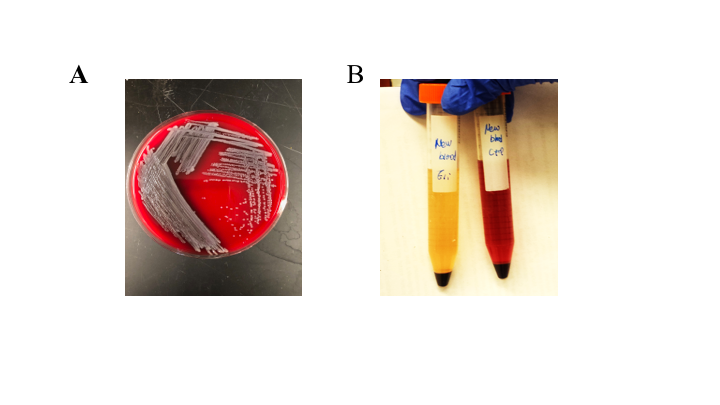
Figure S3. Demonstration of the alpha-hemolysin production and hemoglobin uptake in *E. anophelis*.** A) The greenish colonies on SBA indicated the alpha-hemolytic activity. B) The left tube was inoculated with *E. anophelis* and the right one is control without inoculation. The vials were rotated for overnight and centrifuged.
