# Supplementary material for: Elizabethkingia anophelis: Physiologic and Transcriptomic Responses to Iron Stress
Source: Front Microbiol. 2020 May 7;11:804. doi: 10.3389/fmicb.2020.00804 (PMC7221216; doi:10.3389/fmicb.2020.00804)
Supplement: Supplementary file 4 [file Data_Sheet_1.zip › Figure S4.docx]

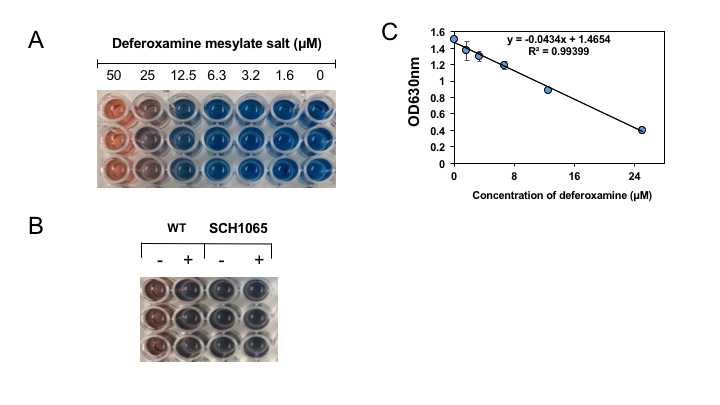


**Figure S4. The siderophore production and determination in WT and mutants of *E. anophelis*.** A) Effects of the adding deferoxamine mesylate on the color change of the iron solution as described in the Methods and Materials. B) The different color change caused by the siderophore production between the WT and the mutant. – low iron condition; + iron rich condition. C) The standard curve for determination of the siderophore production using deferoxamine mesylate.
